# Dietary selenium intake and sarcopenia in American adults

**DOI:** 10.3389/fnut.2024.1449980

**Published:** 2024-09-12

**Authors:** Jianfen Li, Chaohui Jiang, Lingfeng Wu, Jiangyan Tian, Bin Zhang

**Affiliations:** ^1^Department of General Practice, Jiangmen Central Hospital, Jiangmen, China; ^2^Department of Hematopathology, Jiangmen Central Hospital, Jiangmen, China; ^3^Department of Cardiovascular Disease and Clinical Experimental Center, Jiangmen Central Hospital, Jiangmen, China; ^4^Department of Cardiology, The First Affiliated Hospital, Sun Yat-sen University, Guangzhou, China

**Keywords:** dietary selenium intake, sarcopenia, muscle mass, cross-sectional study, National Health and Nutrition Examination Survey

## Abstract

**Background:**

The relationship between dietary selenium intake and sarcopenia remains poorly understood. Therefore, this study investigates the associations between dietary selenium intake and sarcopenia among American adults.

**Methods:**

This cross-sectional study analyzed data from 19,696 participants in the National Health and Nutrition Examination Survey (NHANES) for the periods 1999–2006 and 2011–2018. Appendicular muscle mass, assessed using dual-energy x-ray absorptiometry and adjusted for body mass index, was used as a marker for sarcopenia. Dietary selenium intake was evaluated using the 24-h dietary recall system, and the study accounted for the complex sampling methodology and incorporated dietary sample weights in the analysis.

**Results:**

Among the 19,696 participants, the prevalence of sarcopenia was found to be 8.46%. When compared to the lowest quintile of dietary selenium intake (Q1, < 80.10 μg/day), the odds ratios for sarcopenia in the second quintile (Q2, 80.10–124.61 μg/day) and the third quintile (Q3, >124.61 μg/day) were 0.80 [95% confidence interval (CI): 0.70–0.92, *p* = 0.002] and 0.61 (95% CI: 0.51–0.73, *p* < 0.001), respectively. A negative relationship was observed between dietary selenium intake and sarcopenia (non-linear: *p* = 0.285). Furthermore, sensitivity analyses revealed a robust association between selenium intake and the prevalence of sarcopenia after further adjusting for blood selenium levels.

**Conclusion:**

The results suggest an inverse association between dietary selenium intake and the prevalence of sarcopenia among American adults.

## Introduction

Sarcopenia, a prevalent age-related condition, is characterized by a progressive loss of skeletal muscle mass and function. This decline in muscle physiology leads to diminished physical performance, decreased quality of life, and increased mortality risk (1, 2). The etiology of sarcopenia is multifaceted and not entirely understood, but it is commonly attributed to a combination of factors, including physical inactivity, the aging process, and hormonal imbalances (3). Moreover, nutritional intake plays a crucial role in preserving muscle mass and function throughout life (1).

Selenium, an essential trace element, is vital for normal human body function (4, 5). In mammals, selenium is incorporated into 25 specific selenoproteins as selenocysteine (Sec) (6). Dietary selenium exists in several forms, including selenomethionine, selenocysteine, selenite, and selenate, all of which are efficiently absorbed and have high bioavailability (7). The recommended dietary allowance for selenium in American adults is 55 μg per day (8).

Selenium plays a key role in the synthesis of selenoprotein, a complex process that involves the intracellular recycling of selenium, which is facilitated by selenocysteine metabolism through the action of selenocysteine lyase (9). The synthesis of selenoproteins involves incorporating Sec at UGA stop codons, a process mediated by the Selenocysteine Insertion Sequence (SECIS) located in the mRNA's 3′ untranslated region and SECIS-binding protein 2 (SBP2) (10). Selenophosphate synthetase converts selenide into monoselenophosphate, which is crucial for synthesizing Sec-tRNA [Ser]Sec, ensuring the accurate incorporation of Sec into the growing polypeptide chain (7).

These selenoproteins function as oxidoreductase enzymes and are involved in various metabolic processes (11), including scavenging of free radicals, maintaining intracellular redox balance, and repairing oxidized lipids and methionines. Research indicates that selenoproteins are vital for their musculoskeletal function and may help mitigate the effects of reactive oxygen species, such as hydrogen peroxide (12, 13).

Plasma selenium is a key biomarker for assessing the human selenium status. Although other biofluids such as hair, toenails, and urine can also be analyzed for selenium, plasma testing is most commonly used due to its diagnostic preference (7). Low serum selenium levels have been associated with worse sarcopenic outcomes (14). While dietary intake is the primary source of selenium, research on the association between dietary selenium consumption and adult sarcopenia is limited. Prolonged parenteral nutrition, which can lead to selenium deficiency, has been linked to symptoms such as muscle pain and weakness, which have been resolved with selenium supplementation (15, 16). We hypothesize an inverse association between dietary selenium intake and sarcopenia. To test this hypothesis, we conducted a cross-sectional study to examine the relationship between dietary selenium intake and sarcopenia in American adults.

## Methods

### Survey description and study population

This cross-sectional study utilized data from the National Health and Nutrition Examination Survey (NHANES), covering the periods 1999–2006 and 2011–2018, as managed by the Centers for Disease Control and Prevention (CDC). NHANES aims to assess the health and nutritional status of non-institutionalized Americans. Participants were selected using a stratified probability sampling design involving a multistage process (17). The NHANES dataset encompasses various health metrics, including demographic characteristics, physical examination results, laboratory findings, and dietary habits. Data collection was overseen by the National Center for Health Statistics (NCHS) and adhered to rigorous ethical standards, with informed consent obtained from all participants. The dataset is publicly available through the NHANES website (http://www.cdc.gov/nchs/nhanes.htm). This study included individuals aged 20 years and older who completed the survey. Pregnant women and individuals with incomplete data on dual-energy x-ray absorptiometry (DXA), body mass index (BMI), dietary selenium intake, covariates, or sample weights were excluded from the analysis.

### Assessment of sarcopenia

DXA is highly regarded for measuring body composition due to its speed, ease of use, and minimal radiation exposure (18, 19). From 1999 to 2006, whole-body DXA scans were performed using the Hologic QDR 4500A fan-beam densitometer (Hologic, Inc., Bedford, Massachusetts, USA). From 2011 to 2018, scans were conducted using the Hologic Discovery model A densitometers (Hologic, Inc., Bedford, Massachusetts, USA) with software version Apex 3.2.

Appendicular skeletal muscle mass (ASM) is a critical metric derived from DXA scans, representing the total lean mass of the extremities, including both arms and legs. This measurement is obtained by summing the lean mass, excluding the bone mineral content, provided by DXA scans for these regions. This study assessed sarcopenia using the sarcopenia index, calculated as ASM adjusted by BMI (ASM/BMI). According to the criteria set by the Foundation for the National Institutes of Health (FNIH) Sarcopenia Project (20), men were classified as having sarcopenia if their sarcopenia index was < 0.789 and women were classified as having sarcopenia if their sarcopenia index was < 0.512. These criteria have been employed in recent research (21, 22).

### Assessment of dietary selenium intake

Dietary intake was assessed over 2 separate days in the NHANES study: the initial session was conducted in person, followed by a subsequent session via telephone. Due to significant data gaps encountered during the second round of interviews, our analysis relied solely on the dietary information collected during the initial session. Estimations of dietary selenium and other nutrients were based on data from the Food and Nutrient Database for Dietary Studies (http://www.ars.usda.gov/ba/bhnrc/fsrg), a comprehensive resource provided by the United States Department of Agriculture (23).

### Covariates

A variety of potential covariates were assessed according to the literature (24–32), including age, sex, marital status, race/ethnicity, education level, poverty income ratio (PIR), smoking status, hypertension, diabetes, cardiovascular diseases (CVDs), cancer, physical activity, healthy eating index-2015 (HEI-2015), dietary supplements taken, albumin, estimated glomerular filtration rate (eGFR), uric acid, and total cholesterol. Race/ethnicity was categorized as Non-Hispanic White, Non-Hispanic Black, Mexican American, or other races (26). Marital status was classified into two groups: those who were either married or living with a partner and those who were living alone (26). The educational level was categorized into three groups: less than high school, high school or equivalent, and above high school (27). Family income was divided into three categories based on the PIR: PIR ≤ 1.30, PIR 1.31–3.50, and PIR > 3.50 (27). Smoking status was categorized as never smokers (smoked < 100 cigarettes), current smokers, and former smokers (quit smoking after smoking more than 100 cigarettes) (29). Drinking status was self-reported and categorized as never (had < 12 drinks in a lifetime), former (had ≥12 drinks in 1 year and did not drink last year, or did not drink last year but drank ≥12 drinks in a lifetime), mild (female drinking ≤ 1 and male drinking ≤ 2 per day), moderate (female drinking ≤ 2 and male drinking ≤ 3 per day), or heavy (female drinking ≥3 and male drinking ≥4 per day) (27). The definition of hypertension was self-reported diagnosis, use of antihypertensive drugs, systolic blood pressure ≥140 mmHg, or diastolic blood pressure ≥90 mmHg (24). The definition of diabetes was self-reported diagnosis, use of insulin or oral hypoglycemic agents, fasting glucose ≥7 mmol/L, or HbA1c ≥ 6.5% (31). The CVD history was self-reported as having previously been diagnosed with heart failure, coronary heart disease, angina, heart attack, or stroke (27). The cancer history was self-reported as having been diagnosed with cancer or malignancy. The Healthy Eating Index (HEI) is a robust metric for evaluating the overall quality of an individual's diet, explicitly gauging the alignment of dietary habits with the established Dietary Guidelines for Americans (30). HEI-2015 scores range from 0 to 100, with higher values signifying better diet quality. The Healthy Eating Index (HEI) comprises 13 components that assess compliance with dietary guidelines, including the consumption of the recommended food groups and the limitation of less healthy options. We calculated the HEI scores based on total nutrient intakes from the initial day of dietary assessment, capturing participants' adherence to HEI-2015 standards, which emphasize aspects such as fruit and vegetable intake, whole grains, and reductions in added sugars and saturated fats (30). Dietary supplements were determined by the question regarding nutritional supplements and medications consumed during the past month (29). Furthermore, we quantified the level of physical activity by employing the metabolic equivalent of task (MET), measured in minutes per week (32). Additionally, the eGFR was determined using the Chronic Kidney Disease Epidemiology Collaboration (CKD-EPI) formula, a reliable method for evaluating kidney function.

### Statistical analysis

Our analysis accounted for the complex sampling design and dietary sample weights. For the combined NHANES 1999–2000 and 2001–2002 datasets, we used the 4-year dietary weight (WTDR4YR). We applied the dietary day-one sample weight for the datasets from 2003 to 2004, 2005 to 2006, 2011 to 2012, 2013 to 2014, 2015 to 2016, and 2017 to 2018 (WTDRD1). Sampling weights were calculated as follows: for the 1999–2002 period, weights were set at 1/4 × WTDR4YR, and for other years, they were set at 1/8 × WTDRD1. Categorical data are presented as unweighted counts with weighted percentages, while continuous data are reported as means ± standard deviations (SD). Differences between groups were assessed using one-way analyses of variance for continuous variables and chi-square tests for categorical variables. A multivariate logistic regression analysis was employed to determine the association between dietary selenium intake and sarcopenia, with results expressed as odds ratios (ORs) and 95% confidence intervals (CIs).

Model 1 adjusted for demographic variables, including age, gender, marital status, race/ethnicity, PIR, and education level. Model 2 further accounted for smoking status, alcohol consumption, hypertension, diabetes, CVD history, cancer history, physical activity, HEI-2015, and use of dietary supplements. Model 3 included additional adjustments for albumin, eGFR, uric acid, and total cholesterol.

To evaluate the dose-response relationship between dietary selenium intake and sarcopenia, we employed a restricted cubic spline (RCS) regression model with three knots positioned at the 10th, 50th, and 90th percentiles of dietary selenium intake. This approach allowed us to assess linearity and explore the relationship after adjusting for the covariates in Model 3.

We also performed interaction and subgroup analyses using logistic regression models stratified by age, sex, race/ethnicity, marital status, PIR, smoking status, alcohol consumption, hypertension, diabetes, CVD history, cancer history, and eGFR.

Two sensitivity analyses were conducted to ensure the robustness of our results. Initially, the primary analysis did not include serum selenium due to substantial missing serum selenium data and potential collinearity with dietary selenium. To address whether dietary selenium remains associated with sarcopenia after adjusting for serum selenium, we conducted a sensitivity analysis excluding participants without serum selenium data, followed by a multivariate logistic regression analysis that included serum selenium along with the covariates from Model 3. Multiple imputations with five replications were also used to handle missing covariate data.

Although no prior statistical power calculations were conducted, the large sample size of 19,696 participants provided substantial analytical power. To verify this, we subsequently used G^*^Power software to assess the power of our analyses, which indicated a power of 1. All statistical analyses were performed using R Statistical Software (Version 4.2.2, http://www.R-project.org, The R Foundation) and the Free Statistics analysis platform (Version 1.9.2, Beijing, China, http://www.clinicalscientists.cn/freestatistics). Descriptive statistics were computed for all participants, and statistical significance was evaluated using a two-tailed test with a threshold of *p* of < 0.05.

## Result

### Study population

**Figure 1:**
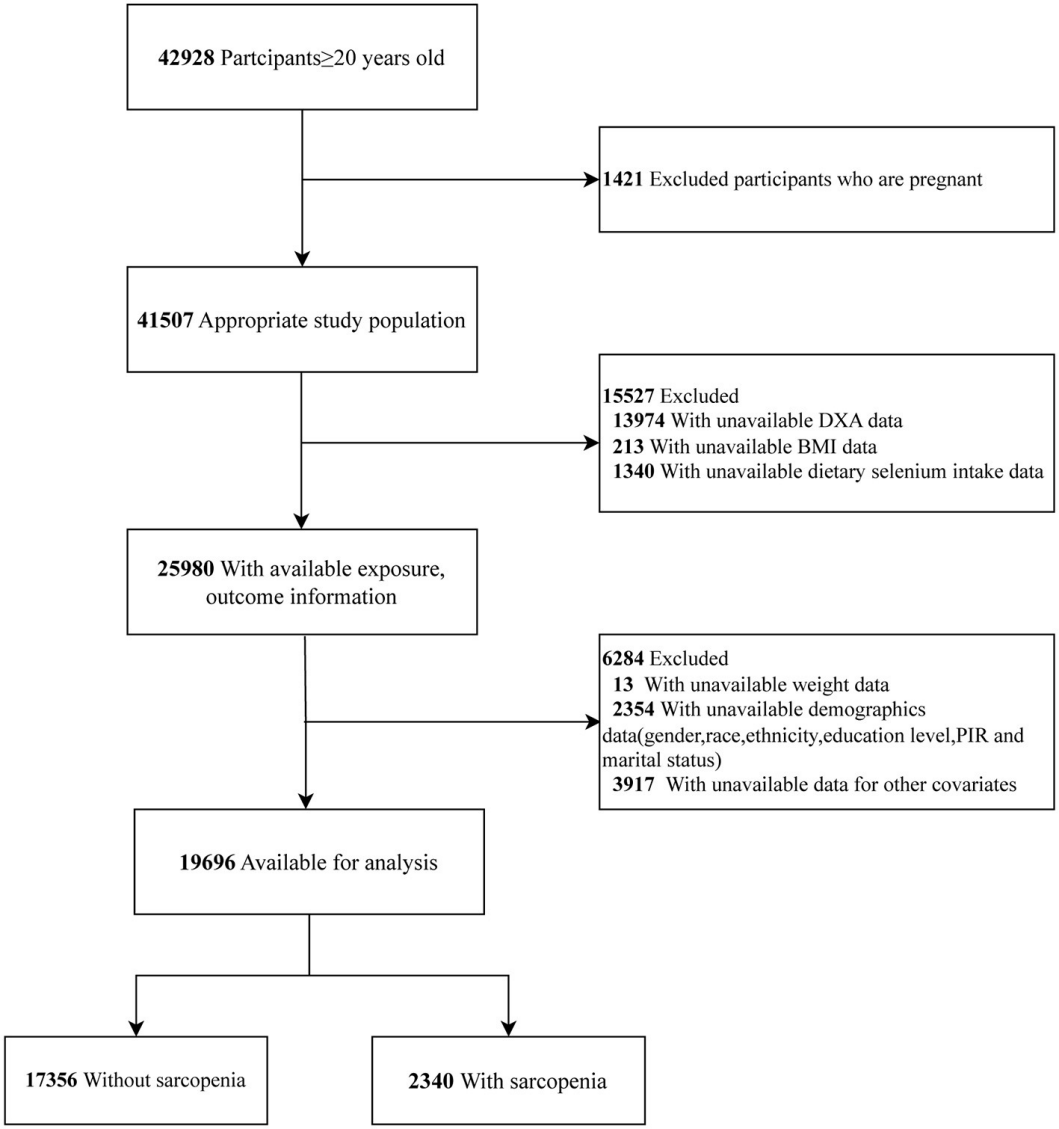
Flow diagram of the screening and enrollment of study participants.

This study utilized data from eight NHANES cycles: 1999–2000, 2001–2002, 2003–2004, 2005–2006, 2011–2012, 2013–2014, 2015–2016, and 2017–2018. A total of 42,928 participants aged 20 years and older completed the survey. We excluded pregnant women (*n* = 1,421) and participants with missing data on DXA (*n* = 13,974), BMI (*n* = 213), and dietary selenium intake (*n* = 1,340). Participants without data on sample weights (*n* = 13) and covariates (*n* = 6,271) were excluded. Consequently, the final sample consisted of 19,696 participants (Figure 1).

### Baseline characteristics

The baseline characteristics of the 19,696 participants with available data for analysis are summarized in Table 1. These participants represent ~124.60 million American adults aged 20 years and older. The mean age was 43.54 (14.68) years, with 9,830 (49.31%) being male. The overall prevalence of sarcopenia was 8.46%. Higher dietary selenium intake was generally observed among younger participants, men, those with a PIR of > 3.5, married or cohabiting individuals, mild alcohol consumers, non-smokers, and those with higher educational attainment. Additionally, higher selenium intake was associated with greater physical activity, higher levels of albumin, uric acid, and eGFR, a lower incidence of hypertension, diabetes, CVD, cancer, and lower total cholesterol levels.

**Table 1:** Baseline characteristics by dietary selenium intake level.

**Characteristics**	**Dietary selenium intake (**μ**g/day)**				***P*-value**
	**Total**	**Q1 (**<**80.10)**	**Q2 (80.10-124.61)**	**Q3 (**>**124.61)**	
	***n*** = **19,696**	***n*** = **6,559**	***n*** = **6,569**	***n*** = **6,568**	
Age, mean (SD), years	43.54 (14.86)	45.58 (15.92)	43.83 (14.96)	41.42 (13.41)	< 0.001
**Sex**, ***n*** **(%)**					< 0.001
Male	9,830 (49.31)	2,194 (30.95)	3,143 (45.85)	4,493 (69.29)	
Female	9,866 (50.69)	4,365 (69.05)	3,426 (54.15)	2,075 (30.71)	
**Race/ethnicity**, ***n*** **(%)**^a^					0.001
Non-Hispanic White	9,167 (69.74)	2,980 (68.94)	3,136 (70.90)	3,051 (69.36)	
Non-Hispanic Black	3,877 (10.24)	1,442 (11.76)	1,252 (9.93)	1,183 (9.17)	
Mexican American	3,715 (8.15)	1,256 (7.47)	1,236 (7.81)	1,223 (9.09)	
Other Hispanic	1,221 (5.36)	393 (5.54)	406 (5.20)	422 (5.36)	
Others^b^	1,716 (6.50)	488 (6.28)	539 (6.17)	689 (7.02)	
**Education level**, ***n*** **(%)**					< 0.001
Less than high school	4,700 (14.87)	1,920 (18.68)	1,454 (13.21)	1,326 (12.99)	
High school or equivalent	4,500 (24.02)	1,521 (25.23)	1,512 (24.34)	1,467 (22.62)	
Above high school	10,496 (61.11)	3,118 (56.09)	3,603 (62.45)	3,775 (64.39)	
**Marital status**, ***n*** **(%)**					0.002
Married or living with a partner	12,261 (64.05)	3,839 (61.48)	4,140 (64.83)	4,282 (64.39)	
Living alone	7,435 (35.95)	2,720 (38.52)	2,429 (35.17)	2,286 (34.37)	
**PIR**, ***n*** **(%)**					< 0.001
≤ 1.3	5,554 (20.82)	2,118 (24.80)	1,755 (19.65)	1,681 (18.31)	
>1.3–3.5	7,428 (34.97)	2,483 (35.65)	2,522 (35.10)	2,423 (34.24)	
>3.5	6,714 (44.21)	1,958 (39.56)	2,292 (45.25)	2464 (47.45)	
BMI, mean (SD), kg/m^2^	28.48 (6.50)	28.30 (6.68)	28.45 (6.41)	28.68 (6.41)	0.062
**Smoking status**, ***n*** **(%)**					0.004
Never	10,766 (53.83)	3,645 (53.59)	3,588 (54.11)	3,533 (53.80)	
Former	4,532 (23.11)	1,424 (21.22)	1,597 (23.96)	1,511 (24.02)	
Now	4,398 (23.05)	1,490 (25.19)	1,384 (21.93)	1,524 (22.19)	
**Drinking status**, ***n*** **(%)**					< 0.001
Never	2,606 (11.11)	1,146 (14.67)	828 (10.77)	632 (8.21)	
Former	3,187 (13.48)	1,248 (15.22)	1,089 (13.94)	850 (11.48)	
Mild	6,491 (34.37)	1,955 (31.16)	2,228 (35.93)	2,308 (35.80)	
Moderate	3,177 (18.26)	1,010 (18.15)	1,133 (18.89)	1,034 (17.31)	
Heavy	4,235 (22.77)	1,200 (20.31)	1,291 (20.47)	1,744 (27.20)	
Hypertension, *n* (%)	7,199 (32.77)	2,653 (35.03)	2,433 (31.86)	2,113 (31.58)	0.005
Diabetes, *n* (%)	2,404 (8.93)	870 (9.66)	856 (9.13)	678 (8.08)	0.039
CVD history, *n* (%)	1,580 (6.37)	672 (7.77)	530 (6.67)	378 (4.81)	< 0.001
Cancer history, *n* (%)	1,307 (7.02)	526 (8.28)	472 (7.96)	309 (4.99)	< 0.001
Physical activity MET, mean (SD), min/week	2,187.82 (4,519.00)	1,783.69 (3,941.14)	2,028.26 (4,227.69)	2,706.68 (5,181.76)	< 0.001
HEI-2015, mean (SD)	51.92 (13.21)	51.77 (13.64)	52.06 (13.03)	51.92 (12.99)	0.662
Dietary supplements taken, *n* (%)	9,524 (52.16)	3,144 (51.98)	3,255 (54.04)	3,125 (50.53)	0.023
ALT, mean (SD), U/L	26.33 (25.24)	23.76 (18.30)	25.70 (21.88)	29.27 (32.37)	< 0.001
AST, mean (SD), U/L	25.27 (17.03)	24.45 (15.83)	25.05 (18.79)	26.22 (16.25)	< 0.001
Albumin, mean (SD), g/dL	4.33 (0.33)	4.29 (0.33)	4.33 (0.33)	4.38 (0.32)	< 0.001
Creatinine, mean (SD), mg/dL	0.87 (0.34)	0.85 (0.39)	0.86 (0.34)	0.90 (0.28)	< 0.001
Uric acid, mean (SD), mg/dL	5.38 (1.40)	5.17 (1.40)	5.32 (1.39)	5.62 (1.36)	< 0.001
Total cholesterol, mean (SD), mg/dL	198.20 (41.46)	199.94 (42.54)	198.59 (40.60)	196.24 (41.20)	0.002
eGFR, mean (SD), mL/(min·1.73 m^2^)	99.38 (19.38)	97.56 (20.88)	99.46 (19.28)	100.96 (17.85)	< 0.001
Sarcopenia, *n* (%)	2,340 (8.46)	987 (10.81)	812 (8.44)	541 (6.35)	< 0.001

Q, quartiles; SD, standard deviation; PIR, poverty income ratio; BMI, body mass index; CVD, cardiovascular disease; MET, metabolic equivalent of task; HEI-2015, healthy eating index-2015; AST, aspartate aminotransferase; ALT, alanine aminotransferase; eGFR, estimated glomerular filtration rate.

Data are presented as unweighted numbers (weighted percentages) for categorical variables, and all means and SDs for continuous variables were weighted.

^a^Race and ethnicity were self-reported.

^b^Includes multiracial participants. NHANES does not provide a detailed list of all races and ethnicities. A *p*-value of < 0.05 was set as the threshold of statistical significance.

### Relationship between dietary selenium intake and sarcopenia

The univariable analysis showed that age, sex, race/ethnicity, education level, PIR, smoking status, alcohol consumption, hypertension, diabetes, CVD, cancer, physical activity, albumin, uric acid, total cholesterol, eGFR, and dietary selenium intake were linked to sarcopenia (Table 2).

**Table 2:** Association of covariates and sarcopenia.

**Covariates**	**OR (95% CI)**	***P*-value**
Age (years)	1.05 (1.04–1.05)	< 0.001
**Sex**		
Female (vs. male)	0.86 (0.75–0.97)	0.018
**Race/ethnicity (vs. Non-Hispanic White)**		
Non-Hispanic Black	0.33 (0.26–0.41)	< 0.001
Mexican American	2.89 (2.48–3.37)	< 0.001
Other Hispanic	1.96 (1.56–2.47)	< 0.001
Others	1.19 (0.91–1.54)	0.195
**Education level (vs. less than high school)**		
High school or equivalent	0.58 (0.50–0.67)	< 0.001
Above high school	0.33 (0.28–0.38)	< 0.001
**Marital status (vs. married or living with a partner)**		
Living alone	0.88 (0.77–1.02)	0.084
**PIR (vs**. ≤ **1.3)**		
>1.3–3.5	0.82 (0.68–0.97)	0.024
>3.5	0.43 (0.37–0.52)	< 0.001
Hypertension (yes vs. no)	2.79 (2.42–3.21)	< 0.001
Diabetes (yes vs. no)	3.82 (3.26–4.48)	< 0.001
CVD history (yes vs. no)	3.66 (3.08–4.35)	< 0.001
Cancer history (yes vs. no)	1.47 (1.15–1.87)	0.002
**Smoking status (vs. never)**		
Former	1.36 (1.16–1.59)	< 0.001
Now	0.79 (0.68–0.93)	0.006
**Drinking status (vs. never)**		
Former	1.15 (0.92–1.44)	0.218
Mild	0.5 (0.40–0.63)	< 0.001
Moderate	0.38 (0.29–0.49)	< 0.001
Heavy	0.48 (0.37–0.62)	< 0.001
Physical activity MET, min/week	1 (1.00–1.00)	< 0.001
HEI-2015	1 (0.99–1.00)	0.163
Dietary supplements taken (yes vs. no)	0.95 (0.82–1.09)	0.456
ALT, U/L	1 (1.00–1.00)	0.135
AST, U/L	1 (1.00–1.01)	0.156
Albumin, g/dL	0.26 (0.22–0.31)	< 0.001
Creatinine, mg/dL	0.87 (0.66–1.15)	0.324
Uric acid, mg/dL	1.2 (1.15–1.26)	< 0.001
Total cholesterol, mg/dL	1 (1.00–1.00)	< 0.001
eGFR, mL/(min·1.73 m^2^)	0.99 (0.99–0.99)	< 0.001
Dietary selenium intake, μg/day	1 (0.99–1.00)	< 0.001
**Dietary selenium intake (vs. Q1**<**80.10** μ**g/day)**		
Q2 (80.10–124.61 μg/day)	0.76 (0.67–0.87)	< 0.001
Q3 (>124.61 μg/day)	0.56 (0.48–0.66)	< 0.001

Q, quartiles; PIR, poverty income ratio; CVD, cardiovascular disease; MET, metabolic equivalent of task; HEI−2015, healthy eating index-2015; AST, aspartate aminotransferase; ALT, alanine aminotransferase; eGFR, estimated glomerular filtration rate; CI, confidence interval; OR, odds ratio.

The unit for continuous variables and the reference group for categorical variables are provided next to the variables. A *p*-value of < 0.05 was set as the threshold of statistical significance.

**Figure 2:**
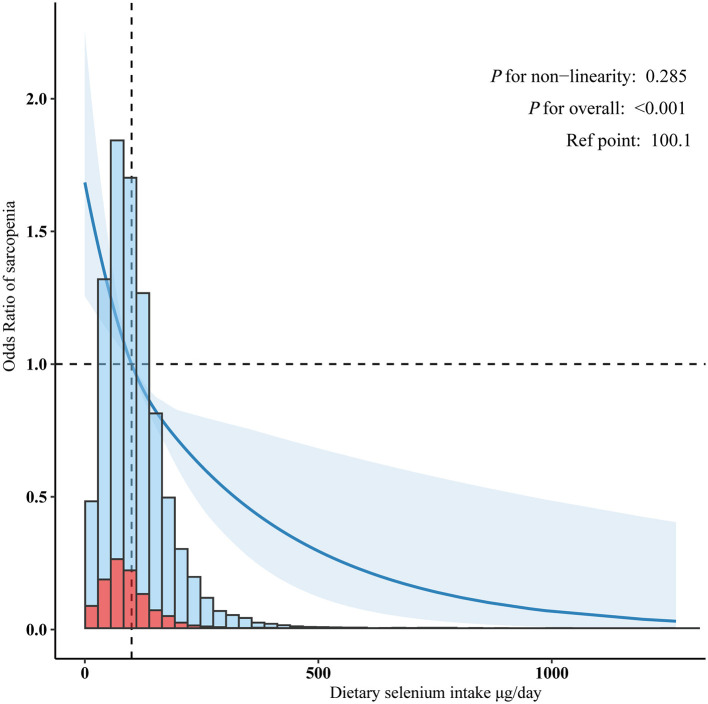
Linear dose-response relationship between dietary selenium intake and sarcopenia. Adjusted for age, sex, poverty income ratio, race/ethnicity, education level, marital status, smoking status, alcohol drinking status, hypertension, diabetes, cardiovascular disease history, cancer history, physical activity, healthy eating index-2015, dietary supplements taken, albumin, estimated glomerular filtration, uric acid, and total cholesterol.

An inverse association between dietary selenium consumption and sarcopenia was observed after adjusting for potential confounders (Table 3). Compared to the lowest dietary selenium intake quintile (Q1, < 80.10 μg/day), the adjusted ORs for sarcopenia in Q2 (80.10–124.61 μg/day) and Q3 (>124.61 μg/day) were 0.80 (95% CI: 0.70–0.92, *p* = 0.002) and 0.61 (95% CI: 0.51–0.73, *p* < 0.001), respectively. Evidence from the estimated dose-response curve indicated a significant linear relationship between dietary selenium intake and sarcopenia (Figure 2, *p* for non-linearity = 0.285).

**Table 3:** Association between dietary selenium intake and sarcopenia.

	**Crude**		**Model 1**		**Model 2**		**Model 3**	

	**OR (95% CI)**	***P*** **-value**	**OR (95% CI)**	***P*** **-value**	**OR (95% CI)**	***P*** **-value**	**OR (95% CI)**	***P*** **-value**
**Dietary selenium intake**								
Q1 (< 80.10 μg/day)	1 (Reference)		1 (Reference)		1 (Reference)		1 (Reference)	
Q2 (80.10–124.61 μg/day)	0.76 (0.67–0.87)	< 0.001	0.82 (0.72–0.94)	0.004	0.83 (0.72–0.95)	0.006	0.8 (0.70–0.92)	0.002
Q3 (>124.61 μg/day)	0.56 (0.48–0.66)	< 0.001	0.64 (0.53–0.76)	< 0.001	0.64 (0.54–0.77)	< 0.001	0.61 (0.51–0.73)	< 0.001
*P* for trend		< 0.001		< 0.001		< 0.001		< 0.001

Model 1: adjusted for age, sex, poverty income ratio, race/ethnicity, education level, and marital status.

Model 2: adjusted for model 1+ smoking status, alcohol drinking status, hypertension, diabetes, cardiovascular disease history, cancer history, physical activity, healthy eating index-2015, and dietary supplements taken.

Model 3: adjusted for model 2+ albumin, estimated glomerular filtration rate, uric acid, and total cholesterol.

Q, quartiles; CI, confidence interval; OR, odds ratio.

A *p*-value of < 0.05 was set as the threshold of statistical significance.

### Subgroup analyses

**Figure 3:**
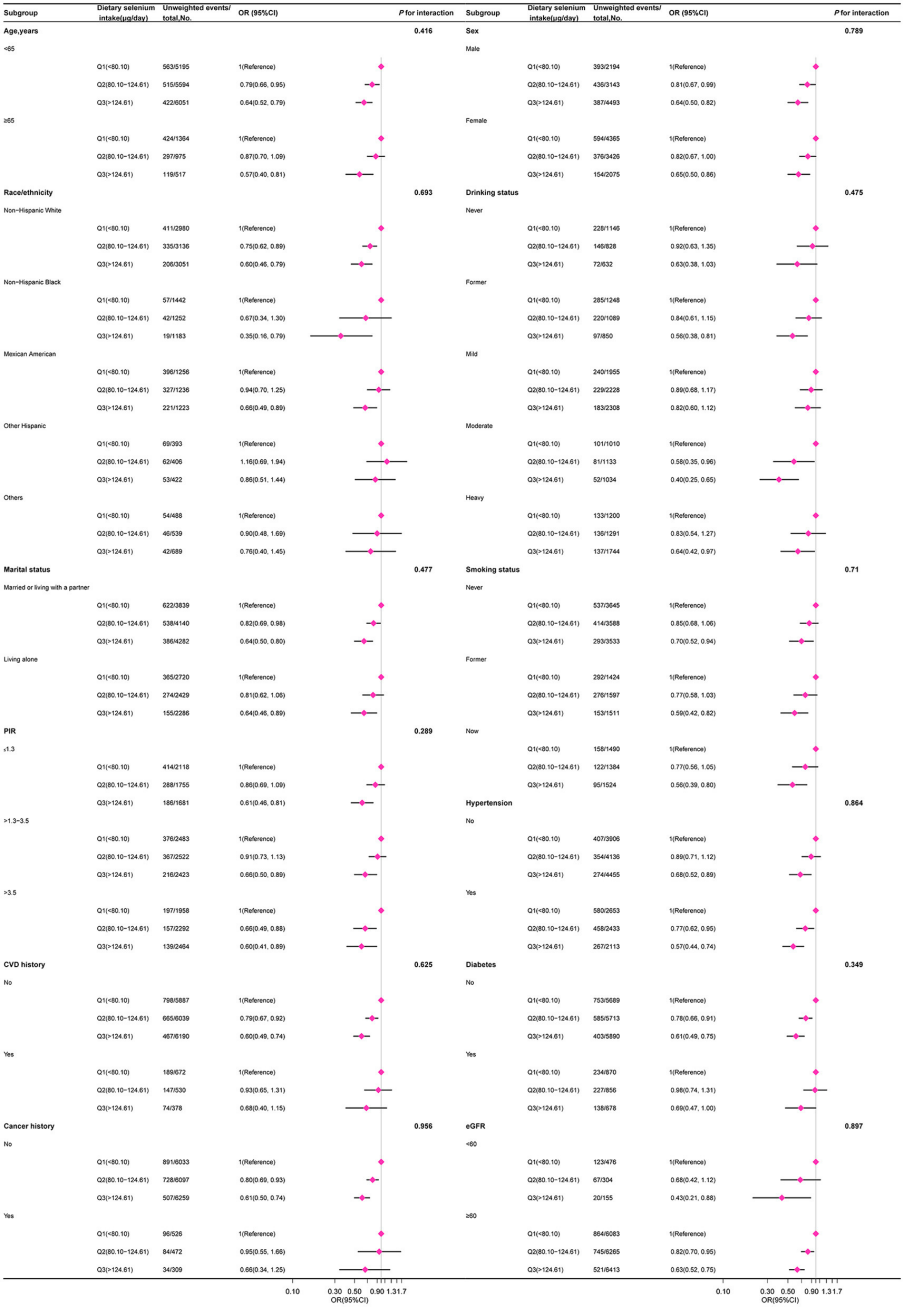
The relation between dietary selenium intake and sarcopenia according to basic features. Except for the stratification component itself, each stratification factor was adjusted for other variables (age, sex, poverty income ratio, race/ethnicity, education level, marital status, smoking status, alcohol drinking status, hypertension, diabetes, cardiovascular disease history, cancer history, physical activity, healthy eating index-2015, dietary supplements taken, albumin, estimated glomerular filtration, uric acid, and total cholesterol).

In several subgroups, a stratified analysis was conducted to assess potential effect modifications on the relationship between dietary selenium intake and sarcopenia. Consistent results were observed when the analysis was stratified by age, sex, race/ethnicity, marital status, PIR, smoking status, alcohol consumption, hypertension, diabetes, CVD, cancer, and eGFR (Figure 3).

### Sensitivity analysis

Initially, participants without serum selenium data were excluded. After further adjusting for serum selenium, the adjusted OR for dietary selenium intake and sarcopenia in Q3 (>124.61 μg/day) was 0.65 (95% CI: 0.43–0.97, *p* = 0.031) compared to the lowest quintile (Q1: < 80.10 μg/day; Table 4). Multiple imputations with five replications were also conducted to address missing covariate data. Out of the 25,980 participants, sample weight data were not available for 13 of them. Multiple imputations were performed for the remaining 25,967 participants. Compared to the lowest quintile (Q1: < 80.10 μg/day), the adjusted OR for dietary selenium intake and sarcopenia in Q3 (>124.61 μg/day) was 0.61 (95% CI: 0.51–0.73, *p* < 0.001; Table 5).

**Table 4:** Sensitivity analyses 1.

**Dietary selenium intake (μg/day)**	**Unweighted events/ total, No**.	**Crude**		**Adjusted** ^**b**^	
		**OR (95% CI)**	***P*** **-value**	**OR (95% CI)**	***P*** **-value**
Total^a^	649/4,284				
Q1 (< 80.10)	273/1,482	1 (Reference)		1 (Reference)	
Q2 (80.10–124.61)	244/1,470	0.92 (0.76–1.11)	0.378	0.87 (0.70–1.09)	0.219
Q3 (>124.61)	132/1,332	0.55 (0.38–0.79)	0.002	0.65 (0.43–0.97)	0.036
*P* for trend			0.001		0.031

^a^Excluding participants with unavailable serum selenium data.

^b^Adjusted for age, sex, poverty income ratio, race/ethnicity, education level, marital status, smoking status, alcohol drinking status, hypertension, diabetes, cardiovascular disease history, cancer history, physical activity, healthy eating index-2015, dietary supplements taken, albumin, estimated glomerular filtration, uric acid, total cholesterol, and serum selenium.

Q, quartiles; CI, confidence interval; OR, odds ratio. A *p*-value of < 0.05 was set as the threshold of statistical significance.

**Table 5:** Sensitivity analyses 2.

**Dietary selenium intake (μg/day)**	**Unweighted events/ total, No**.	**Crude**		**Adjusted** ^**b**^	
		**OR (95% CI)**	***P*** **-value**	**OR (95% CI)**	***P*** **-value**
Total^a^	3,100/25,967				
Q1 (< 80.10)	1,346/8,842	1 (Reference)		1 (Reference)	
Q2 (80.10–124.61)	1,038/8,509	0.75 (0.66–0.86)	< 0.001	0.77 (0.68–0.89)	< 0.001
Q3 (>124.61)	716/8,616	0.58 (0.49–0.68)	< 0.001	0.61 (0.51–0.73)	< 0.001
*P* for trend			< 0.001		< 0.001

^a^Multiple imputations of missing data.

^b^Adjusted for age, sex, poverty income ratio, race/ethnicity, education level, marital status, smoking status, alcohol drinking status, hypertension, diabetes, cardiovascular disease history, cancer history, physical activity, healthy eating index-2015, dietary supplements taken, albumin, estimated glomerular filtration, uric acid, and total cholesterol.

Q, quartiles; CI, confidence interval; OR, odds ratio. A *p*-value of < 0.05 was set as the threshold of statistical significance.

## Discussion

This cross-sectional study identified an inverse relationship between dietary selenium intake and sarcopenia. Stratified and sensitivity analyses further confirmed a robust association between dietary selenium and sarcopenia in American adults. Extensive research suggests that selenium may play a role in musculoskeletal health, with recent studies linking reduced serum selenium levels to exacerbated sarcopenic symptoms (33–37). Despite this finding, evidence regarding the relationship between dietary selenium intake and sarcopenia remains limited and inconsistent. For example, the Newcastle 85+ Study (28) found that low selenium intake was associated with diminished musculoskeletal function in participants aged 85 years and older. Conversely, other studies did not observe a significant relationship between dietary selenium and musculoskeletal health (38–41). Our study contributes to understanding this relationship by demonstrating an inverse association between dietary selenium intake and sarcopenia in American adults, highlighting the need for further prospective research to clarify the effects of selenium on sarcopenia.

According to the dietary reference intakes established by the Institute of Medicine, the recommended daily allowance for selenium in adults is 55 μg (8). Selenium is sourced from various dietary items, including meats, seafood, cereals, grains, dairy products, fruits, and vegetables. Our study population, consisting of individuals aged 20 years and older, generally exceeds this recommended intake. Therefore, the current recommendation for selenium intake might be insufficient. Some research suggests that the existing recommended daily intake may not be adequate (42). However, excessive selenium intake can lead to toxicity (43), although the World Health Organization considers up to 400 μg per day to be safe (44). Thus, further research is needed to determine the optimal daily selenium intake.

Selenium is well-regarded for its role in enhancing muscle function, particularly due to its antioxidant properties. Selenoproteins, such as glutathione peroxidase (GPx), are crucial for neutralizing reactive oxygen and nitrogen species, which support muscle health (45–48). These antioxidants are essential for countering oxidative stress, which typically increases with age due to diminished antioxidant defenses (49, 50). The high oxygen demand of skeletal muscle results in the substantial production of reactive nitrogen species, which are associated with reduced muscle strength and mass, often due to increased protein breakdown and decreased muscle protein synthesis (51).

In addition to its antioxidant functions, selenium affects muscle function through other mechanisms. Research indicates that selenium can enhance mitochondrial biogenesis and improve mitochondrial function in skeletal muscle. This effect is partly due to its involvement with various selenoproteins beyond GPx. For instance, selenoprotein H promotes mitochondrial biogenesis, while selenoproteins N and W influence calcium homeostasis in muscles, impacting mitochondrial function (52–54). Additionally, the presence of selenoprotein O in mitochondria suggests that it may facilitate selenium's redox functions within these organelles (55).

The relationship between selenium and muscle function involves complex regulatory mechanisms, including mitochondrial energetics and cellular signaling pathways (56). Given these diverse roles, selenium is crucial for maintaining muscle function and mitigating age-related declines often linked to oxidative damage and impaired mitochondrial performance. Future longitudinal studies are needed to elucidate the causal mechanisms underlying selenium's effects on muscle function.

The strength of our study lies in its use of a sophisticated multistage probability sampling design combined with rigorous covariate adjustment, which enhances the reliability and representativeness of the findings. Nonetheless, several limitations should be noted. First, the study was conducted exclusively with American adults aged 20 years and older, which may limit the generalizability of the results to other demographic groups. Second, the accuracy and validity of the nutritional assessments were constrained using the 24-h dietary recall method. While food frequency questionnaires were not used due to their provision of less detailed information on specific foods and quantities consumed, their inclusion might have provided additional context (57, 58). Finally, the cross-sectional design of the study prevents concluding causality. Future research using prospective cohort studies is essential to elucidate the cause-and-effect relationship between dietary selenium intake and sarcopenia.

## Conclusion

This study identified an inverse relationship between dietary selenium intake and sarcopenia in the adult American population, suggesting that selenium may be a crucial nutrient in influencing the risk of sarcopenia. Based on these findings, we recommend that public health nutritional guidelines incorporate recommendations for adequate selenium intake as a potential strategy to mitigate the risk of sarcopenia. Further longitudinal and interventional studies are essential to establish a causal relationship between selenium intake and sarcopenia and to elucidate the underlying mechanisms. Additionally, research is needed to determine the optimal daily selenium intake for maximizing health benefits.

## Data Availability

Publicly available datasets were analyzed in this study. This data can be found at: https://www.cdc.gov/nchs/nhanes.
