# Supplementary material for: Dietary selenium intake and sarcopenia in American adults
Source: Front Nutr. 2024 Sep 12;11:1449980. doi: 10.3389/fnut.2024.1449980 (PMC11426168; doi:10.3389/fnut.2024.1449980)
Supplement: Supplementary file 1 [file Table_1.DOCX]

**Supplementary Materials Files**

To:**Dietary Selenium Intake and Sarcopenia in American Adults**

by Jianfen Li, Chaohui Jiang, Lingfeng Wu, Jiangyan Tian, Bin Zhang

**Correspondence:** Bin Zhang zhangb87@mail2.sysu.edu.cn

Supplementary Files 1. Table S1. Baseline characteristics of patients with and without serum selenium data.

Table S1. Baseline characteristics of patients with and without serum selenium data.

| Variables | Total (n = 25967) | With serum selenium data  (n = 5324) | without serum selenium data  (n = 20643) | *p-*value |
| --- | --- | --- | --- | --- |
| Sex, n (%) |  |  |  | 0.89 |
| Male | 12986 (50.0) | 2667 (50.1) | 10319 (50) |  |
| Female | 12981 (50.0) | 2657 (49.9) | 10324 (50) |  |
| Age, Mean (SD), years | 45.2 ± 16.5 | 50.5 ± 17.1 | 43.8 ± 16.1 | < 0.001 |
| Race/ethnicity, n (%) ^b^ |  |  |  | < 0.001 |
| Non-Hispanic White | 11392 (43.9) | 2516 (47.3) | 8876 (43) |  |
| Non-Hispanic Black | 5399 (20.8) | 1012 (19) | 4387 (21.3) |  |
| Mexican American | 5013 (19.3) | 911 (17.1) | 4102 (19.9) |  |
| Other Hispanic | 1735 (6.7) | 353 (6.6) | 1382 (6.7) |  |
| Others | 2428 (9.4) | 532 (10) | 1896 (9.2) |  |
| Education level, n (%) |  |  |  | 0.08 |
| Less than high school | 6667 (25.7) | 1303 (24.5) | 5364 (26) |  |
| High school or equivalent | 5981 (23.1) | 1245 (23.4) | 4736 (23) |  |
| Above high school | 13296 (51.2) | 2771 (52.1) | 10525 (51) |  |
| Marital status, n (%) |  |  |  | 0.461 |
| Married or living with a partner | 15565 (60.9) | 3267 (61.4) | 12298 (60.8) |  |
| Living alone | 9978 (39.1) | 2056 (38.6) | 7922 (39.2) |  |
| PIR, n (%) |  |  |  | 0.672 |
| ≤1.3 | 7070 (29.5) | 1467 (29.4) | 5603 (29.5) |  |
| >1.3-3.5 | 9031 (37.6) | 1852 (37.2) | 7179 (37.8) |  |
| >3.5 | 7895 (32.9) | 1663 (33.4) | 6232 (32.8) |  |
| BMI^,^ Mean (SD), kg/m^2^ | 28.6 ± 6.6 | 28.8 ± 6.4 | 28.6 ± 6.6 | 0.101 |
| Smoking status, n (%) |  |  |  | < 0.001 |
| Never | 14227 (54.8) | 2838 (53.3) | 11389 (55.2) |  |
| Former | 5753 (22.2) | 1389 (26.1) | 4364 (21.2) |  |
| Now | 5966 (23.0) | 1095 (20.6) | 4871 (23.6) |  |
| Drinking status, n (%) |  |  |  | < 0.001 |
| Never | 3339 (13.6) | 740 (14.5) | 2599 (13.4) |  |
| Former | 3896 (15.9) | 994 (19.5) | 2902 (14.9) |  |
| Mild | 7938 (32.4) | 1650 (32.4) | 6288 (32.4) |  |
| Moderate | 3900 (15.9) | 745 (14.6) | 3155 (16.3) |  |
| Heavy | 5433 (22.2) | 964 (18.9) | 4469 (23) |  |
| Physical activity MET, mean (SD), min/week | 2195.7 ± 4802.8 | 2378.3 ± 4869.8 | 2148.6 ± 4784.4 | 0.002 |
| HEI-2015, mean (SD) | 52.1 ± 13.2 | 54.0 ± 13.1 | 51.6 ± 13.1 | < 0.001 |
| Hypertension, n (%) | 9384 (36.1) | 2310 (43.4) | 7074 (34.3) |  |
| Diabetes, n (%) | 3145 (12.1) | 795 (14.9) | 2350 (11.4) |  |
| Sarcopenia, n (%) | 3100 (11.9) | 787 (14.8) | 2313 (11.2) |  |
| CVD history, n (%) | 2091 (8.1) | 629 (11.8) | 1462 (7.1) |  |

SD, standard deviation; PIR, poverty income ratio; BMI, body mass index; CVD, cardiovascular disease; MET, metabolic equivalent of task; HEI-2015, healthy eating index-2015; Race and ethnicity were self-reported. c Includes multiracial participants. NHANES does not provide a detailed list of all races and ethnicities. *P* < 0.05 was set as the threshold of statistical signiﬁcance.
